# Is the FIFA 11+ Warm-Up Effective for Inducing Acute Knee Adaptations in Recreational Soccer Players?

**DOI:** 10.3390/jfmk10020216

**Published:** 2025-06-05

**Authors:** Patricia Caudet, Ernest Baiget, Abraham Batalla, Joshua Colomar, Miguel Crespo, Rafael Martínez-Gallego, Francisco Corbi

**Affiliations:** 1 National Institute of Physical Education (INEFC), University of Lleida, 25192 Lleida, Spain; patriciacaudet@gmail.com; 2 National Institute of Physical Education (INEFC), University of Barcelona, 08038 Barcelona, Spain; 3 Sport Science Group Research of National Institute of Physical Education (INEFC), University of Barcelona, 08038 Barcelona, Spain; 4 EUSES School University of Health and Sport, Rovira I Virgili University, 43870 Tarragona, Spain; gavalda13@gmail.com; 5 Department of Education and Specific Didactics, Faculty of Humanities and Social Sciences, University Jaume I, 12071 Castellón de la Plana, Spain; 6 Sports and Physical Activity Studies Center (CEEAF), University of Vic-Central University of Catalonia, 08500 Vic, Spain; joshua.colomar@uvic.cat; 7 Sports Performance Analysis Research Group (SPARG), University of Vic-Central University of Catalonia, 08500 Vic, Spain; 8 Development Department, International Tennis Federation, London SW15 5XZ, UK; miguel.crespo@itftennis.com; 9 Department of Physical Education and Sport, University of Valencia, 46010 Valencia, Spain; rafael.martinez-gallego@uv.es; 10 Department of Clinical Sciences, Faculty of Medicine and Health Sciences, University of Barcelona, 08907 L’Hospitalet de Llobregat, Spain; f@corbi.neoma.org

**Keywords:** warm-up, knee, soccer, prevention, injury, FIFA11+, football

## Abstract

**Objectives:** Soccer is the most practiced sport around the world. The injury incidence has an estimated rate of up to 70 injuries per 1000 h of play. FIFA 11+ is a program designed to prevent injuries and optimize performance. The purpose of this study was to analyze the acute effects of this program as a warm-up on different functional, physiological, and mechanical properties of various knee tissues and whether there were differences between genders. **Methods**: The sample included 45 recreational soccer players. Several muscular and tendon mechanical properties, muscular oxygen saturation, electromyography, maximum voluntary contraction, and rate of force development were analyzed, before and after performing the FIFA 11+. **Results**: Only a moderate significant increase in muscle oxygen saturation in men from pre- to post-test was reported. No other parameters showed statistically significant differences between groups, suggesting that the intervention may lack clinical relevance. The reported effect sizes were mostly trivial, so differences are unlikely to have significant practical relevance. Statistical analyses were performed using a 2 × 2 factorial repeated measures factorial ANOVA with Bonferroni post hoc comparisons. **Conclusions**: FIFA 11+ warm-up does not provide a sufficient stimulus to elicit mechanical or metabolic responses in the per-knee structures. Other warm-up designs may be more appropriate for finding these effects.

## 1. Introduction

Soccer is the most famous and practiced sport worldwide. An estimated 200,000 professional and 240 million amateur players practice soccer every weekend [1]. In the United States, soccer was the sport with the highest number of student-athlete participants at both the high school and collegiate levels [2]. Moreover, match-play requires high-intensity actions (approximately 600 actions and about 40 high intensity actions over 21 km/h) where running, jumping and ball striking [3] patterns are applied with high inertial loads and large accelerations, which causes a high incidence of injuries. Specifically, an estimated rate of up to 70 injuries per 1000 h of play has been reported [1]. Furthermore, the risk of injury appears to be greater during competition (24.29/1000 h) than in training (6.84/1000 h) [4]. Consequently, injuries among male professional players incur in a cost of approximately 204,206 € per 1000 h playing hours [5], and 500,000 € per month [6]. These high costs must be assumed by soccer clubs, leagues, and sports federations, having a high impact on their economies. This is particularly relevant for recreational soccer players, who represent the largest group at risk and are often underserved in terms of injury prevention resources.

The most common injuries affect the lower limbs (70–80%) [7] and among the most affected body areas the knee stands out, both for quantity and severity [5]. For example, around 66% of former players suffered knee injuries [8] and 47.5% of total injuries involved the knee, being the body region that requires highest surgical intervention rates (60%) [9]. Furthermore, high rates of radiographic osteoarthritis in the knees of former professional players have been observed [10], probably due to the overload placed on this joint during their careers [11]. For this reason, it has been considered that the practice of soccer could have significant negative effects on the knee joint [7]. Added, some of the soccer injuries that require the longest recovery time occur in the knee. The total rupture of the ACL provokes an absence from soccer for 7–12 months [12] and represents, for professional players, a drop in their maximum performance that is typically not achieved until five years later [13]. Future problems are associated with this injury such as osteoarthritis, a high rate of new injuries to the graft or the opposite knee, and even abandonment of the sports career [13,14]. Most recently, in the FIFA world cup Qatar 2022 [15], the most frequent body area injured was the thigh, with hamstring muscle injuries being most frequent (19% of all injuries). For all these reasons, although other anatomical regions such as the hamstrings and ankles also present high injury rates in soccer, the knee stands out not only for its frequency but also for the severe functional and economic consequences associated with its injuries [5,6,16]. Furthermore, many preventive interventions have shown contradictory results specifically regarding this joint [16]. Medical staff, physiotherapists, and trainers should take measures to prevent the appearance of knee-related injuries. Prevention programs [17] as strengthening training, stretching exercises [18] or warm-up protocols [19] could be interesting strategies to implement in order to avoid injuries. Systematic reviews and meta-analyses question the effectiveness of multicomponent exercise-based injury prevention programs, claiming to be inconclusive across all ages and genders, reflecting heterogeneity or a lack of statistical power [20,21]. However, these strategies reduced overall and ACL injuries in women, 27% and 45%, respectively [20]. Despite this, there is still limited knowledge regarding the acute local effects of these interventions on specific joints such as the knee—particularly when considering potential sex differences.

Warm-up protocols seem to reduce injury risk through the improvement of muscle temperature, blood flow, oxygen consumption, nerve conductivity, muscle activation and force expression, which allows the player to improve physiological parameters and focus, self-confidence and performance moving players away from the injury threshold [22]. For example, muscle strength can be improved by 3.46–4.21% and speed and jumping by 1%–20% acutely after specific warm-ups [18,23]. Strength and power capabilities can be represented on the force–time continuum, which may reflect adaptations of the neuromuscular system to the training process. Therefore, tests such as the isometric mid-thigh pull (IMTP) are used [24]. Furthermore, understanding the excitability of certain muscles can be very helpful in understanding the athlete’s activation pattern and the movement requirements. Electromyography (EMG) details these patterns and allows for more precise task preparation. Other programs, such as dynamic load warm-up programs (DLWU), improved change in direction performance (CODS) in badminton athletes up to 18 min after performance, possibly due to the PAP effect induced by repeated SSC on Achilles tendon stiffness, which allows muscles to more effectively resist external forces, reducing energy expenditure [25]. The patellar tendon (PT) acts as a force-transmitting tendon that improves performance in actions such as walking, running, jumping, and squatting [25,26,27]. Non-invasive digital palpation tools (NIRS) such as the MyotonPro^®^ (MyotonPRO, Myoton AS, Tallinn, Estonia) are tools that assess stiffness in tendons and muscles. Other NIRS such as the Humon Hex (Humon Hex device, Dynometrics Inc., Cambridge, MA, USA.), monitor muscle oxygenation status, indicating fatigue and muscle performance thresholds during and after the task.

Pre-match warm-up is considered a main strategy for injury prevention in soccer [13] as it stimulates the components that make up the joints that are most influential in the sport, favoring the athlete’s performance and reducing the likelihood of injury. Unfortunately, in amateur soccer, its application is not feasible due to the lack of existing economic resources [14], which does not allow having formed staff to correctly carry out a well-rounded warm-up. For this reason, the Fédération Internationale de Football Association (FIFA) created a warm-up program that could reduce the risk of typical injuries in soccer called FIFA11+. Its structure is based on the combination of cardiovascular, neuromuscular, and cognitive exercises with a duration of around 20–25 min [28], focusing on central stability, eccentric and plyometric work, proprioception, and dynamic stability [28].

Although FIFA11+ seems to reduce the incidence of lower extremity injuries [29] and increase the performance [30], the recent literature has shown a great divergence of opinions about its local effects. While some studies seem to show how FIFA+11 seems to improve knee stability, maximum knee abduction moment, quadriceps eccentric strength and muscle activation (sartorius, semimembranosus, biceps femoris, and hallux muscles) [31], others find no significant improvements in knee muscle strength [32]. Even so, there are few studies that focus on the acute effects on local joints, especially the knee. Warm-up should facilitate not only improvements in physical performance but also the optimization of local functionality of the joints involved during sports activities, particularly in regions with a high incidence of injuries such as the knee. Physiological and mechanical responses to warming up may be modulated by sex-gender differences such as hormonal production and regular hormonal cycles. For example, women generally present a differential distribution of body fat percentage, with thicker adipose tissue in the extremities and a lower percentage of type II fibers, which implies a lower capacity to generate force and a greater capacity to maintain body temperature [33]. It is hypothesized that these variations influence the magnitude and nature of the acute effects of the FIFA+11 protocol on the biomechanical and physiological properties of the knee since the incidence of knee injuries is higher in women than in men [6]. This lack of evidence represents a significant research gap that warrants further investigation. Therefore, the primary objective of this study was to analyze the acute effects of FIFA+11 as a pre-match warm-up on various functional, physiological, and mechanical properties of different knee tissues in male and female recreational players. It also sought to determine whether these effects differ between genders.

## 2. Materials and Methods

### 2.1. Experimental Approach to the Problem

**Figure 1:**
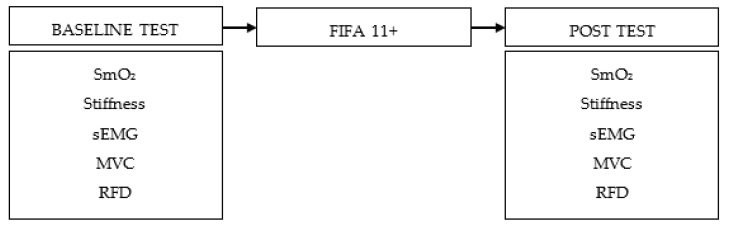
Study design timeline. SmO_2_ = muscle oxygen saturation, sEMG = Surface electromyography, MVC = maximum voluntary muscle contraction, RFD = force development ratio.

A descriptive observational study with pre-post analyses was developed. The independent variable was associated with the FIFA 11+ warm-up, and dependent variables included muscular and tendon mechanical properties, muscular oxygen saturation (SmO_2_), electromyography (EMG), maximum voluntary contraction (MVC), and rate of force development (RFD) (Figure 1). The study design consisted of two collecting sessions. Baseline tests were recorded prior to performing the warm-up. After, the tests were repeated in the second session. All tests were analyzed in the dominant extremity (leg used to kick). Test order was randomized to prevent physical and psychological fatigue influence within each session. All tests were performed in the absence of fatigue. The second session was carried out just after the warm-up finished to prevent the effects of the warm-up from decreasing over time. Participants did the protocol between 9am and 4pm and in similar weather conditions.

### 2.2. Participants

A total of 45 recreational football players, 24 male (age 22.7 ± 2.9 years, height 177.3 ± 6.0 cm; body mass 73.7 ± 7.4 kg; mean ± SD) and 21 females (age 21.9 ± 1; height 164.5 ± 6.1; body mass 58.3 ± 8.0 kg; mean ± SD) participated in this study (Table 1). The sample size was justified by a priori power analysis (using GPower Version 3.1.9.5, University of Dusseldorf, Dusseldorf, Germany) for a repeated measures (within-between interaction) introducing the following parameters: effect size index (0.25) assuming a medium partial eta-squared (0.06), α error probability (0.05), power (0.90), number of groups (2) and measurements (2), and correlation among repeated measurements (0.5) which resulted in a sample size of 45 subjects. Participants were recruited from some Catalonia universities (Catalonia, Spain). Inclusion criteria were related to sports practice 2 times a week. Exclusion criteria were related to previous lower extremity injury in the last 6 months. Also, they were instructed to continue their lifestyle and normal diet during previous days. Nobody received economic or species benefits to participate in this study. Further, they signed an informed consent, and a protocol was established for the delivery and explanation of the results. However, the voluntary right to leave the study is also addressed, in case the participants ultimately did not want to be admitted without prejudice or harm and a guarantee of their anonymity. The study was approved by the Bioethics committee from Barcelona University (Institutional Review Board IRB00003099 CER042418). This study was carried out considering the principles of the Declaration of Helsinki for research with human beings (AMM, 2013) and in accordance with the Data Protection Law (BOE.es-BOE-A-2018-16673 Organic Law 3/2018, of December 5, on the Protection of Personal Data and guarantee of digital rights).

### 2.3. Procedures

#### 2.3.1. Warm-Up FIFA 11+

It is composed of three parts: (a) 6 slow velocity running exercises combined with active stretching and contact with a partner; (b) 6 progression exercises aimed at strength, plyometrics, agility and balance; (c) 3 running exercises of moderate to high speed with changes in direction. These parts have a 20–25 min duration [34].

#### 2.3.2. Mechanical Properties

A non-invasive digital palpation device MyotonPro^®^ (MyotonPRO, Myoton AS, Tallinn, Estonia) was used to test individual muscle response. Body marks were established using the SENIAM electrode placement guides [35]. The mechanical properties analyzed were: frequency (F, Hz) (intrinsic mechanical tension in passive or resting state); stiffness (resistance to contraction or external force that modifies a muscle’s initial shape); decrement (muscle elasticity in terms of the recovery of the muscle’s initial state or removal of a tensile stress); time to relaxation (time it takes for the muscle to return to its initial state after being deformed by an external force); creep (gradual elongation of the muscle over time when a constant external force is applied) [36]. The muscle groups chosen were as follows: 1. Biceps femoris at the midpoint between the ischial tuberosity and the fibular head [37]. 2. Rectus femoris at the midpoint between the anterior superior iliac spine and the upper part of the patella [38]. 3. Patellar tendon selected midway between the distal part of the patella and the tibial tuberosity [38]. In all measurements, the tip of the MyotonPro^®^ was placed perpendicular to the relaxed muscle tissue [38] with the end of the device located at 3 mm [39] with a short mechanical impulse (0.4 N) sent by the device for 15ms. An average between five ratings at 3200 Hz was calculated [39]. The MyotonPro^®^ reliability shows excellent test–retest values (ICC = 0.852 to 0.993) [37].

Figure SEQ Figure\* ARABIC 1. Chronology of tests carried out. RPE: rate perceived exertion; HR: heart rate; SmO_2_: muscle oxygen saturation; Tsk: skin temperature; WBLT: Weight Bearing Dorsiflexion Lunge Test; MVIC: maximum voluntary isometric contraction; KS: Kick speed; CMJ: Countermovement squat jump.

#### 2.3.3. Muscular Oxygen Saturation (SmO_2_)

The oxygen muscular saturation was analyzed using a non-invasive near-infrared spectroscopy (NIRS) [40], (Humon Hex device, Dynometrics Inc., Cambridge, MA, USA.). Its light detectors located at 18–24 mm with a sample measurement of 4 Hz, reflecting the value of the weighted median [40]. This device was placed on medial rectus femoris like MyotonPro^®^ and was recorded for 30 s, while the player was sitting with 90° flexion in hip and knee. It is connected by Bluetooth to the Moxzones application mobile device. Its reliability was indicated with an ICC = 0.77–0.99 [40].

#### 2.3.4. Surface Electromyography (sEMG)

sEMG is designed to assess motor plate muscle activity in superficial muscles. The mDurance^®^ system (mDurance Solutions SL, Granada, Spain) is a portable, low-cost sEMG device that consists of three parts: (a) Shimmer3 EMG unit (Realtime Technologies Ltd., Dublin, Ireland), bipolar sEMG sensor for obtaining superficial muscle activity where each sensor has two channels with a sampling frequency of 1024 Hz. Shimmer applies a bandwidth of 8.4 Hz with a resolution of the EMG signal of 24bits and an overall amplification of 100–10,000 V/V. The electrodes are pre-gelled Ag/AgCl with a diameter of 10 mm and a distance between them of 20 mm. (b) mDurance^®^ mobile application (Android) where the Shimmer signal is received, and the data is sent to the cloud service. (c) Cloud service that stores, filters, and analyzes electrical signals generating a report [41]. The recommendations of the SENIAM guidelines were followed for the preparation of the skin and the location of the electrodes [35] on the biceps femoris and rectus femoris with a reference electrode on the fibula head. Data was collected during the MVC and RFD tests. Its reliability was indicated with an ICC > 0.81 [41].

#### 2.3.5. Maximum Voluntary Force (MVC) and Force Development Ratio (RFD)

The protocol proposed by Colomar et al. (2022) [42] was followed. The exercise was performed using an 80 Hz strain gauge attached to a weighted metal base and a barbell placed between the thighs, simulating a portable force platform. For subsequent analysis, appropriate software (Chronojump, Boscosystem, Barcelona, Spain) was used. Participants were instructed to pull the barbell as hard and quickly as possible once positioned on the base or a weighted metal plate. The mid-thigh position was determined before the test by marking the distance from the midpoint between the knee and hip joints, specifically between the iliac crest and the patella. The barbell height was adjusted for each athlete to ensure contact with the mid-thigh. A pronated or hook grip was permitted. The effort was maintained for 5 s. Participants completed a total of three trials, with a 30 s rest interval between trials, and the mean value was selected for analysis. MVC and peak RFD were defined as the maximum value reached during the 5 s period. The RFD was then calculated as the segment between the time points followed by the equation: RFD = ΔForce/ΔTime. Accuracy and reproducibility were assessed using a test–retest design, in which participants performed two trials with 2 min of rest between trials, with one trial being randomized to avoid the influence of fatigue. Measurements showed acceptable levels of reliability and good to excellent intraclass correlation coefficients (ICC ≥ 0.823) across all variables [42].

### 2.4. Statistical Analyses

The values presented are expressed as mean ± SD. The normality of the distributions and homogeneity of variances were assessed with the Kolmogorov–Smirnov test. Differences between sexes were compared using an independent sample *t*-test. Independent variables were defined in terms of the different gender (male and female) and the 2 measurement time points (pre- vs. post-test). The dependent variables were the physiological and kinematic parameters. Data were analyzed using a 2 × 2 factorial repeated–measures ANOVA with Bonferroni’s post hoc comparisons, using one between-group factor (male or female) and one within-group factor (pre- vs. post-test). The partial eta-square (h^2^) effect size was calculated to evaluate the main and interaction effects of ANOVA. The h^2^ values of 0.01–0.05, 0.06–0.13, and >0.14 indicate small, medium, and large effect sizes, respectively. To determine the magnitude of within-group change in variables, a Cohen’s d effect size (ES) was performed on pairwise comparisons. The criteria to interpret the magnitude of the ES were 0.0–0.2 trivial, 0.2–0.6 small, 0.6–1.2 moderate, 1.2–2.0 large, and >2.0 very large. Twenty pre-planned comparisons were considered for this study. Accordingly, correction for multiple comparisons was undertaken using the Bonferroni method with a resulting operational alpha level of 0.0025 (*p* = 0.05/20). Intrasession reliability of measures was determined using a 2-way average measure of the intraclass correlation coefficient (ICC) and coefficient of variation (CV). The ICC values were interpreted as follows: excellent (>0.85), high (0.75–0.85), moderate (0.40–0.75), and poor (<0.40). Statistical significance was accepted at an alpha level of *p* < 0.05. All statistical analyses were performed using IBM SPSS Statistics 23.0 software (SPSS, Inc., Chicago, IL, USA).

## 3. Results

Changes in the selected physiological and kinematic variables from pre- to post-warm-up for each gender are reported in Table 2. After completing the standardized warm-up, only a large main effect of time was found in muscle oxygen saturation (F [1.166] = 18.86; *p* < 0.001; h^2^ = 0.305). The post hoc test showed moderate significant increases in muscle oxygen saturation in men from pre- to post-test (*p* < 0.001; **Δ** = 14.3%; ES = 0.66). No significant gender x time interactions were observed for any physiological and kinematic variable. The corresponding effect sizes were mostly trivial, suggesting that these differences are unlikely to be meaningful in practical terms.

## 4. Discussion

The primary objective of this study was to evaluate the acute local effects of the FIFA 11+ protocol on biomechanical and physiological variables of the knee in male and female soccer players. Most of the parameters analyzed showed no statistically significant differences between groups, suggesting that the evaluated intervention does not have a clinically relevant impact on these variables. Furthermore, the reported effect sizes were mostly trivial, indicating that the observed differences are unlikely to have significant practical relevance. From this perspective, FIFA 11+ does not offer substantial benefits in the conditions studied regarding knee joint outcomes. However, moderate effect sizes were observed in relation to muscle oxygen saturation, which could indicate potentially relevant effects in certain contexts or specific subgroups. Numerous studies have demonstrated the effectiveness of FIFA 11+ in improving performance and preventing injuries in amateur footballers. From a global perspective, FIFA 11+ seems to avoid injuries thanks to preserving athletes from potentially risky movement patterns [31], reducing peak knee valgus moment during jumps [43], knee valgus collapse during side cuts [44], and increasing static and dynamic body balance [34,44] and coordination [45]. Contrarily, others research did not find any improvements in fundamental movement patterns [46] or in pre-planned cutting movements [34,46]. This variability in results may be related to a lack of standardization in experimental design, such as program duration, weekly frequency, and participant adherence and characteristics. Also, some studies focus on specific biomechanical variables, while others evaluate physiological or general performance parameters, which can lead to different conclusions about the program’s effectiveness. The methodological quality of the studies often varies; some present a high risk of bias due to a lack of randomization or blinding. Part of these discrepancies are due to differences in how FIFA 11+ is implemented: longer and more intensive interventions tend to show greater benefits, while shorter or poorly implemented programs present less consistent results [46].

On the other hand, these discrepancies could be explained because in the FIFA 11+ program the exercises applied are mainly static, particularly in part 2, where many tasks with a low level of intensity and neuromuscular function [46] are involved. Dynamic warm-up should increase local muscle temperature and blood supply which results in a higher anaerobic metabolic capacity and peripheral nervous conduction [47], thanks to an increased blood supply, both globally and locally. Therefore, the type of exercise selected and the intensity at which it is performed seem to be critical to stimulate peripheral local adaptations. In our study, our working hypothesis was that a warm-up that improves functional movement does not necessarily improve response in local body regions. This could happen because the warm-up could activate neuro-muscular response [48], thanks to the stimulation of the corticospinal axons, changes in motoneuron’s spinal-level excitability and an increased brain [49] activation before other brain cortical areas [50]. Moreover, the effects could be different depending on the gender of the participants, because cerebral circulation in females seems to be influenced by reproductive hormones [51]. For these reasons, in our study, we decided to evaluate local acute responses in the knee from a dual perspective, biomechanical (sEMG, MF, RFD, IMP and muscle mechanical properties) and physiological (SmO_2_) point of view, trying to evaluate knee local acute response in male and female football players. Most of the analyzed parameters did not show statistically significant differences between groups, suggesting that the evaluated intervention may not have a clinically relevant impact on these variables. Furthermore, the reported effect sizes were mostly trivial, indicating that the observed differences are unlikely to have significant practical relevance. From this perspective, FIFA11+ might not offer substantial benefits in the conditions studied regarding knee joint outcomes. However, moderate effect sizes were observed in relation to muscle oxygen saturation, which could indicate potentially relevant effects in certain contexts or specific subgroups. Therefore, further analyses would be advisable to identify whether certain patient profiles might benefit more from the intervention.

From a biomechanical perspective, warm-up routines should increase post-activation potentiation (PAP), increasing the capacity to generate strength and improving the efficiency of muscle contraction, thanks to a simultaneous increase in firing rate and the number of motor units recruited, especially for the high-threshold motor units [52]. These effects should translate into a gain in the ability to generate greater amounts of force production in small periods of time, thanks to an increase in the pinna angle and the recruitment of fast twitch muscle motor units and in the phosphorylation of myosin regulatory light chains [25]. In our study, RFD (rate of the rise in force over time) [53], maximal force (MF) and impulse in the Isometric Mid-Thigh Pull (IMTP) test were selected as strength indicators. Previously, numerous studies used this test to predict strength performance in soccer players where they found differences in the force production of the knee flexors after 8 weeks of intervention in young soccer players [24,54]. Instead, the results obtained in our study suggest that the FIFA11+ protocol does not have sufficient potential to generate changes in the force–time curve variables analyzed, which calls into question its applicability in improving performance in certain strength variables. Understanding this provides insight into how the impulse–momentum theorem develops [55], allowing us to assess the velocity variability required to design specific exercises similar to the demands of competition, as 74% of injuries occur at high or moderate horizontal movement speeds [56]. A lack of variability in this parameter prevents the metabolic–hormonal and mechanical changes necessary for competition.

In addition, in our research, FIFA11+ was also an insufficient stimulus to generate significant changes in tone, stiffness, and elasticity related to key muscles and the patellar tendon, except for the activation of the biceps femoris in females, which is in fact noteworthy as hamstrings strains represent the most common injury in soccer [57]. This could be due to many reasons. First, FIFA 11+ does not align with the principle of dynamic correspondence [58]. In the Nordic Hamstring Exercise (NHE) high levels of biceps femoris activation in this exercise has been observed previously, which suggests that it may still provide a powerful stimulus for adaptation [59] in female players. In contrast, males did not experience any variation after the application of the FIFA11+. These could be explained because previous activation levels were lower in women than in men, which would imply that lower levels of intensity were needed to obtain changes [60] after the warm-up protocol. NHE seems to induce significant delayed onset muscle soreness (DOMS) [46,61,62] in players, which has been shown to have negative effects prior to competition and can impair performance [63]. Impellizzeri et al. (2021) [64] questioned the efficacy of the NHE as a strategy for injury risk prevention, while Cuthbert et al. (2020) [62] and Amundsen et al. (2022) [65] suggest reducing its application in terms of training volume. On the other hand, Della Villa et al. (2023) [56] proposed exercises involving perturbations during horizontal actions such as running as a preventive method, since it is under these circumstances that a high number of injuries occur.

Warm-up protocols should produce changes in local mechanical properties. Exercise should cause an increase in the stiffness levels of tendons and muscles. For example, recently, Nguyen et al. (2025) [66] observed how running 6 min at 65% of maximal aerobic speed generated an increase in the stiffness of the Achilles tendon and gastrocnemius muscles, and Paravlic et al. (2024) [67] confirmed a positive contractile response when tested with tensiomyography between pre- and post- warm-up in basketball players. In our study, the FIFA11+ protocol was not able to generate changes in the local mechanical responses of the tissues studied, possibly because of the type of exercise used and the intensities at which the exercises were performed. The current literature suggests that exercises with low volume (short duration and few repetitions) and moderate-to-high intensity when designing tasks to be performed during warm-ups [63]. More specifically, Della Villa et al. (2023) [56] suggested incorporating specific warm-up exercises that develop maximum velocities in open- and closed-chain kinetic actions. For this reason, the level of intensity developed may not have been enough to stimulate strength and muscular or tendinous tissues, which questions the usefulness of the FIFA11+ program to provide adequate intensity of stimulus to prevent associated injuries in these specific tissues [24,68,69].

From a physiological point of view, in our study, we analyzed muscular oxygen saturation of the rectus femoris. Many studies highlight the influence of muscle oxygenation on kinematic and kinetic muscle response [70] since an increase in local blood supply and temperature should translate into an increase in performance [71]. This is because warm-ups should increase oxygen delivery to the muscles via a right-ward shift in oxyhaemoglobin dissociation curve and vasodilatation [72]. In our study, FIFA11+ increased local SmO_2_ muscular oxygen saturation with no differences between sexes, suggesting that the warm-up applied improves local blood supply in the same way in men and women. These results contradict the findings of other authors that claim that men have a greater reduction in SmO_2_ in the locomotor muscles, due to a greater contraction speed and muscle area, which causes a greater oxygen consumption and a greater recruitment of IIx fibers [73]. Also, Sendra et al. (2023) [74] explained how men have a greater dependence on oxygen extraction. In the case of women, due to their higher percentage of I fibers and greater capillary density in the peripheral muscles, they are provided with a better supply of oxygen that allows them to sustain muscular work with a greater capacity for oxygen consumption and thus obtain a higher SmO_2_ [73,74]. This could be related to better vasodilation mediated hormonally by estrogens that activate mechanisms of increased stimulation of endothelial potassium channels, reduced superoxide anion production or upregulation of superoxide dismutase, and improved endothelium-dependent relaxation by increasing nitric oxide production/release, or decreased degradation [74,75]. However, in Sendra’s study, when the load of the test was increased, the values on the vastus lateralis in SmO_2_ were indifferent between genders. This greater proportion of I fibers and greater capacity to redistribute or supply oxygen to the exercised tissue could be the reason why women maintain their SmO_2_ levels compared to men. On the other hand, results obtained could be different depending on the muscle studied because previous investigations analyzed power-generating muscles with a high percentage of anaerobic fibers as vastus lateralis. Recent studies demonstrated how vastus medialis’ response depends mainly on fascicle length more than muscle volume and type II fibers, especially when considering the main role of type I fibers at shortening velocities [76]. Another factor to consider could be the influence of adipose tissue (ATT) on the near-infrared spectroscopy (NIRS) instruments, since it can vary the values obtained. ATT is directly related to sex, where women tend to have a higher proportion of this variable, which is at the same time inversely related to the level of performance [74]. However, as no differences were obtained in our study, further investigations should be developed to clarify what factors could influence SmO_2_ and its role in mechanical muscle and tendon response.

Since FIFA11+ appears to have no impact on local body regions, several strategies are proposed to improve it. First, the cluster sets, which allow for greater expression of speed and power with a lower mechanical and metabolic response. Their design would be related to low volumes at medium-high intensities. If these clusters are performed in specific environments, they will increase neuromuscular performance, causing less loss of execution speed, less accumulated fatigue, and fewer metabolic and hormonal alterations [56,77]. Therefore, horizontal actions in open and closed kinetic chains are essential. Another recommendation could be to develop a PAPE effect using hip abduction and external rotation exercises to counteract the muscular weakness of the muscles involved in these movements, which promote the development of ACL injuries [13,16].

This study presented some limitations. First, the lack of a control group makes it difficult to attribute changes (or lack thereof) exclusively to the intervention, as other factors could be influencing the process. In the female sample, menstrual cycle was not recorded, which means that the heating warm-up effect could vary depending on natural hormonal fluctuations. These fluctuations can alter values such as laxity, strength, body temperature, and neuromuscular control [78], changing mechanical response and increasing injury rates. For example, in elite female futsal players, different rates of muscle injury have been observed depending on the phase of the menstrual cycle, suggesting a possible relationship between hormonal fluctuations and a higher injury risk [79]. All this could indicate that warm-up effects could vary depending on the moment of the menstrual cycle. Despite this limitation, our study is the first approximation to understand the local influence the FIFA 11+ has on the knee. In real-life contexts, female players must compete every weekend regardless of their menstrual cycle. Based on the results obtained in this study, future research should analyze the relationship between the players’ menstrual cycle phase and warm-up effects. Second, as myotonometry could not internally register the mechanical response of the muscles and tendons, in our study, we assume that the results obtained are representative of the internal tissue response. The Myotonometer provides a ratio of the amount of tissue including skin, subcutaneous fat, and muscle that is displaced directly underneath the probe to the amount of force applied to the tissue [80]. Despite this, previous studies used this type of technique in the assessment of the mechanical properties of quadriceps femoris and patellar tendon at various angles of knee flexion with excellent inter-operator reliability (ICC > 0.78) and good to excellent inter-operator reliability (ICC > 0.41) [26]. In the same way, other studies demonstrated good intrasession (ICC > 0.807) and interrater (ICC > 830) reliability and moderate intersessions (ICC < 0.693) reliability in hamstrings [80] and from good to excellent reliability (ranging from 0.87 to 0.98) in tone, stiffness, and creep of hamstring aponeuroses. On the other hand, the possible influence of the test order on the results was not considered, despite their randomization. We acknowledge that we did not analyze whether the order in which these tests were performed could have had any impact on the results. Since the study’s objective was to identify acute effects derived from FIFA 11+, long-term effects were not evaluated. Therefore, we cannot determine whether the results measured in the medium-to-long term may have a lasting impact on athletes’ performance. Furthermore, these results are only applicable to athletes of the same level as the study (recreational athletes), as the effects could differ in different population groups. However, numerous studies have focused on the use of FIFA 11+ as a training protocol, with long-term follow-up. While results vary, it remains an effective tool for injury prevention and performance improvement in this area of study [29,43,44]. Another consideration is the limitation of laboratory assessments, which may not fully capture how an individual performs in real-life or sporting situations, due to their artificial and less specific environment for practical activity. This can affect the applicability and generalizability of the results to real-life contexts. Another relevant individual factor is circadian chronotype. Endocrine studies have shown that individuals with an early circadian phenotype present different hormonal profiles than those with evening phenotypes, particularly in terms of morning cortisol levels and diurnal hormonal rhythms. Since cortisol is essential for muscle function, a circadian offset could negatively affect physical performance during certain times of the day. Therefore, it would be advisable to consider hormonal variations associated with individual circadian rhythms in future studies [81]. Finally, a Bonferroni correction was applied to account for multiple comparisons, which reduced the operational alpha to 0.0025. While this correction is considered conservative, it was applied to reduce the likelihood of false positives, given the number of comparisons made. Importantly, the main findings remained consistent regardless of the correction: *p*-values were either significantly lower or higher than both the corrected and uncorrected thresholds.

## 5. Conclusions

In conclusion, the results of our study suggest that, in recreational football players, the FIFA11+ protocol does not significantly enhance acute mechanical properties of periarticular muscles and tendons or maximal isometric strength around the knee joint. However, an improvement in muscle oxygen saturation (SmO_2_) was observed in both sexes, indicating a positive effect on local tissue oxygenation. These findings should be interpreted with caution due to key limitations of our study: the absence of a control group and the exclusive inclusion of recreational athletes, which restricts the generalizability of our results to elite or professional soccer players.

The results highlight the need to develop more specific warm-up protocols adapted to the physiological and mechanical demands of periarticular tissue. To this end, future research should explore strategies such as post-activation potentiation (PAP), enhanced post-activation potentiation (PAPE), and the use of cluster sets, which maximize speed and power output through controlled volumes and medium-high intensities, minimizing fatigue. Implementation of these approaches could facilitate more favorable acute responses in local tissue properties, contributing to improved athletic performance and injury prevention. 

## Figures and Tables

**Table 1:** Descriptive characteristics of participants.

	Sex Groups		Total (*n* = 45)
	Male (*n* = 24)	Female (*n* = 21)	
Chronological Age (years)	22.71 ± 2.85	21.86 ± 0.96	22.31 ± 2.20
Height (cm)	177.33 ± 6.01 *	164.49 ± 6.14	171.34 ± 8.83
BM (kg)	73.70 ± 7.44 **	58.28 ± 7.96	66.50 ± 10.88
BMI (kg·m^−2^)	23.45 ± 2.32 **	21.44 ± 1.81	22.52 ± 2.30

The values presented are means ± SD. BM, body mass; BMI, body mass index. * significantly different from female group (*p* < 0.001). ** significantly different from female group (*p* = 0.001).

**Table 2:** Pre- and post-warm-up physiological and kinematic characteristics scores.

	Gender Groups								Two-Way ANOVA	
	Women (*n* = 21)				Men (*n* = 24)				Time Effect	Time X Gender Effect
	Pre	Post	Δ (%)	ES	Pre	Post	Δ (%)	ES	*p*-Value	
**SmO_2_**										
Mean (%)	52.7 ± 8.3	57.2 ± 10.5 §	8.5	0.38	60.0 ± 6.8 **	68.6 ± 7.2 §ǂ	14.4	1.02	<0.001	0.176
**IMTP**										
MAXFOR (N·s^−1^)	743.6 ± 94.5	741.4 ± 103.2	−0.3	−0.09	1126.6 ± 214.9	1112.1 ± 192.2	−1.3	−0.31	0.156	0.293
RFD (N·s^−1^)	2906.3 ± 1067.8	2991.0 ± 1158.9	2.9	0.12	3341.7 ± 1608.5	3176.0 ± 985.2	−5.2	−0.10	0.835	0.521
IMP (N·s^−1^)	267.0 ± 67.9	256.6 ± 60.1	−4.0	−0.24	378.9 ± 79.1	361.4 ± 103.1	−4.8	−0.27	0.1	0.667
**EMG**										
**Biceps Femoris**										
RMS (µV)	45.2 ± 19.6	52.5 ± 24.4	16.4	0.73	53.5 ± 25.2	52.1 ± 22.1	−2.7	−0.09	0.127	0.028
MCV (µV)	175.3 ± 123.0	179.9 ± 140.4	2.6	0.03	183.0 ± 87.3	132.7 ± 62.2	−37.9	−0.64	0.276	0.192
**Rectus Femoris**										
RMS (µV)	15.2 ± 11.1	15.0 ± 8.8	−0.9	−0.02	11.5 ± 6.6	12.8 ± 9.0	11.5	0.14	0.657	0.588
MCV (µV)	58.8 ± 42.5	59.9 ± 31.8	1.9	0.03	69.3 ± 39.9	57.3 ± 34.5	−21.0	−0.22	0.441	0.356
**MUSCULAR PROPERTIES**										
**Biceps Femoris**										
Frequency (Hz)	14.4 ± 1.2	14.5 ± 1.3	1.0	0.17	16.0 ± 1.6	15.9 ± 1.8	−0.7	−0.10	0.912	0.413
Stiffness (N/m)	242.1 ± 30.2	248.7 ± 29.8	2.7	0.30	295.3 ± 39.5	292.9 ± 46.8	−0.8	−0.07	0.646	0.323
Decrement (*n*.u.)	1.10 ± 0.1	1.1 ± 0.2	−1.2	−0.15	1.2 ± 0.2	1.1 ± 0.1	−2.8	−0.23	0.201	0.623
Relaxation (ms)	21.7 ± 2.6	21.3 ± 2.1	−1.7	−0.22	18.0 ± 2.3	18.4 ± 2.3	2.2	0.21	0.976	0.161
**Rectus Femoris**										
Frequency (Hz)	14.4 ± 1.3	14.4 ± 1.2	−0.2	−0.03	16.0 ± 1.0	15.9 ± 1.4	1.1	−0.13	0.559	0.691
Stiffness (N/m)	259.9 ± 37.0	255.2 ± 34.9	−1.8	−0.19	294.7 ± 26.3	292.2 ± 38.6	−0.9	−0.09	0.386	0.792
Decrement (*n*.u.)	1.5 ± 0.2	1.4 ± 0.2	−2.1	−0.16	1.5 ± 0.3	1.4 ± 0.3	−5.9	−0.32	0.105	0.452
Relaxation (ms)	21.8 ± 2.7	21.9 ± 2.2	0.5	0.06	18.7 ± 1.5	19.1 ± 2.3	1.7	0.16	0.466	0.708
**Patellar Tendon**										
Frequency (Hz)	19.0 ± 2.2	18.7 ± 2.9	−1.5	−0.14	22.6 ± 5.5	22.4 ± 5.4	−0.9	−0.10	0.431	0.876
Stiffness (N/m)	475.5 ± 116.8	444.3 ± 138.1	−7.0	−0.32	596.7 ± 212.5	582.9 ± 194.6	−2.4	−0.17	0.101	0.52
Decrement (*n*.u.)	1.2 ± 0.2	1.2 ± 0.2	−0.3	−0.03	1.2 ± 0.1	1.2 ± 0.1	−3.5	−0.43	0.21	0.297
Relaxation (ms)	12.2 ± 2.6	13.0 ± 2.9	6.5	0.38	10.7 ± 3.3	10.5 ± 3.4	−1.4	−0.11	0.228	0.079

The values presented are means ± SD. Time and time x group interaction *p*-values were obtained by two-way ANOVA (2 gender groups x 2 times). SmO_2_ = muscle oxygen saturation, IMTP = isometric mid-thigh pull, EMG = Electromyography, RMS = root mean square, MVC = maximum voluntary muscle contraction. Bonferroni > 0.0025 different to post-test values. Intragroup significant differences form pre- to post-test: § *p* < 0.001 different to pretest values. Differences between genders in the pre- and post-test: ** Pre-test differences between women (*p* < 0.001). ǂ Post-test differences between women (*p* < 0.001).

## Data Availability

The data will be deposited in CORA-RDR for a minimum period of 10 years. Institutional repository: The data will be deposited in CORA-Repositori de Dades de Recerca (https://dataverse.csuc.cat/dataverse/UdL), a federated and multidisciplinary data repository that allows Catalan universities, CERCA research centers and other research entities to publish research datasets in FAIR mode and following EOSC guidelines. The repository assigns a unique access URL for the data, assigning a DOI, for persistent identification and citability of the dataset. Data are available upon reasonable request.

## Abbreviations

The following abbreviations are used in this manuscript:

SmO_2_	Muscular oxygen saturation
NIRS	Near-infrared spectroscopy
HSI	Hamstring strain injuries
DOMS	Delayed onset muscle soreness
NHE	Nordic Hamstring Exercise
RFD	Force development ratio
MF	Maximal force
IMTP	Impulse in the Isometric Mid-Thigh Pull
PAP	Post-activation potentiation
MVC	Maximum voluntary force
sEMG	Surface electromyography
EMG	Electromyography
FIFA	Fédération Internationale de Football Association
SSC	Stretch-shortening cycle
